# Nuclear ubiquitin proteasome degradation affects WRKY45 function in the rice defense program

**DOI:** 10.1111/tpj.12035

**Published:** 2012-11-08

**Authors:** Akane Matsushita, Haruhiko Inoue, Shingo Goto, Akira Nakayama, Shoji Sugano, Nagao Hayashi, Hiroshi Takatsuji

**Affiliations:** Disease Resistant Crops Research Unit, National Institute of Agrobiological Sciences2–1–2 Kannondai, Tsukuba, Ibaraki, 305–8602, Japan

**Keywords:** salicylic acid, proteasome, rice, transcription factor, WRKY, NPR1, *Arabidopsis thaliana*, *Oryza sativa*

## Abstract

The transcriptional activator WRKY45 plays a major role in the salicylic acid/benzothiadiazole-induced defense program in rice. Here, we show that the nuclear ubiquitin–proteasome system (UPS) plays a role in regulating the function of WRKY45. Proteasome inhibitors induced accumulation of polyubiquitinated WRKY45 and transient up-regulation of WRKY45 target genes in rice cells, suggesting that WRKY45 is constantly degraded by the UPS to suppress defense responses in the absence of defense signals. Mutational analysis of the nuclear localization signal indicated that UPS-dependent WRKY45 degradation occurs in the nuclei. Interestingly, the transcriptional activity of WRKY45 after salicylic acid treatment was impaired by proteasome inhibition. The same C-terminal region in WRKY45 was essential for both transcriptional activity and UPS-dependent degradation. These results suggest that UPS regulation also plays a role in the transcriptional activity of WRKY45. It has been reported that AtNPR1, the central regulator of the salicylic acid pathway in Arabidopsis, is regulated by the UPS. We found that OsNPR1/NH1, the rice counterpart of NPR1, was not stabilized by proteasome inhibition under uninfected conditions. We discuss the differences in post-translational regulation of salicylic acid pathway components between rice and Arabidopsis.

## Introduction

In response to pathogen attacks, plants activate defense systems that are mediated through various signaling pathways. One such system is the salicylic acid (SA) defense signaling pathway, which is mediated by the endogenous signaling molecule SA. The role of SA in systemic acquired resistance has been extensively studied in dicots (Sticher *et al*., 1997; Durrant and Dong, 2004; Loake and Grant, 2007). Exogenous application of SA or its functional analogs such as benzothiadiazole (BTH) induces SA pathway-mediated defense responses in plants, leading to strong disease resistance. Generally, constitutive activation of defense responses is at the expense of plant growth and development (Heil and Baldwin, 2002; Heidel *et al*., 2004; Heidel and Dong, 2006). Therefore, plants have presumably developed mechanisms to suppress unnecessary defense responses so that their growth is not compromised. The SA/BTH-induced defense response involves extensive reprogramming of the plant transcriptome. It is most likely that transcription factors that play roles in this process are under elaborate regulation to avoid the costs of defense responses.

In Arabidopsis, the transcriptional co-factor NPR1 (AtNPR1), which contains ankyrin repeats and the BTB/POZ domain, plays a central role in regulating the SA/BTH pathway. Its loss-of-function mutant *npr1* shows a severely compromised SA/BTH-induced defense response (Delaney *et al*., 1995). Genome-wide transcript profiling revealed that more than 99% of BTH-responsive genes are AtNPR1-dependent (Wang *et al*., 2006). A change in the intracellular localization of AtNPR1 is critical for its function (Kinkema *et al*., 2000; Mou *et al*., 2003; Tada *et al*., 2008). In the absence of a pathogen, AtNPR1 is localized in the cytoplasm as oligomers. Upon attack by pathogens or application of SA/BTH, AtNPR1 converts from an oligomer to a monomer and is translocated into the nucleus, where it interacts with transcription factors (TFs) such as TGAs to transcriptionally activate defense genes (Despres *et al*., 2000). Recently, Spoel *et al*. (2009) showed that AtNPR1 undergoes degradation by the ubiquitin–proteasome system (UPS) in the nucleus. It has been proposed that AtNPR1 is regulated by the UPS in two ways: first, the UPS constitutively degrades AtNPR1 to suppress spurious activation of the defense response in the absence of pathogen attack, and second, SA-induced degradation of AtNPR1 by the UPS results in full-scale activation of the transcriptional activity of AtNPR1.

The WRKY family of transcription factors regulates diverse physiological processes in plants by binding to W-box elements in target promoters, thereby affecting transcription of downstream genes (Eulgem *et al*., 1999; Miao *et al*., 2004; Zou *et al*., 2004; Luo *et al*., 2005; Eulgem and Somssich, 2007; Ren *et al*., 2010). Many members of this family have been implicated in defense against pathogens in Arabidopsis, rice and other plant species. In Arabidopsis, several WRKY transcription factors are regulated downstream of AtNPR1 and mediate SA/BTH signaling (Wang *et al*., 2006). Each plays either a positive or a negative role in regulating plant defenses against pathogens in response to the SA/BTH signal (Wang *et al*., 2006). However, many of these studies were based on phenotypic and gene expression profiling analyses; thus, there is limited information about post-translational regulation of WRKY transcription factors.

Rice has a high level of endogenous SA, which does not appear to increase upon pathogen infection (Silverman *et al*., 1995). Nevertheless, chemical inducers such as BTH and probenazole, which act on the SA pathway in dicots, induce resistance to pathogens such as fungal blast and bacterial leaf blight. Probenazole also induces SA accumulation in rice in an age-dependent manner (Iwai *et al*., 2007). Previously, we identified a BTH-inducible transcription factor WRKY45, which plays an essential role in BTH-induced blast resistance (Shimono *et al*., 2007). Rice plants over-expressing *WRKY45* showed extremely strong resistance to fungal blast (Shimono *et al*., 2007) and bacterial leaf blight (Shimono *et al*., 2012). WRKY45 belongs to group III of the WRKY family (Eulgem *et al*., 2000; Kalde *et al*., 2003), and its likely ortholog in Arabidopsis is WRKY70 (Li *et al*., 2004; Wu *et al*., 2005). Rice has an AtNPR1 counterpart, OsNPR1/NH1, which also plays a crucial role in the SA/BTH pathway (Chern *et al*., 2005). Epistasis analysis showed that the SA/BTH pathway of rice branches into WRKY45-dependent and OsNPR1-dependent pathways, unlike that of Arabidopsis, in which the AtNPR1-dependent pathway is predominant. Genome-wide transcriptome analysis showed that OsNPR1 plays a somewhat limited role compared with AtNPR1 (Sugano *et al*., 2010). Thus, rice has an SA-dependent signaling pathway whose regulatory mechanism(s) appears to differ substantially from that in dicots. However, the details of this regulatory mechanism remain unclear.

In this study, we characterized the post-translational regulation of WRKY45 to deepen our understanding of the function and regulation of WRKY45 in the rice SA signaling pathway. We found that WRKY45 was regulated by UPS-mediated protein degradation in the nucleus. The proteasomal degradation appeared to occur constitutively. Pathogen-mimicking treatments transiently suppressed proteasomal degradation, suggesting that this degradation has a role in preventing spurious defense activation in the absence of pathogen attack. In addition, our results suggested that WRKY45 degradation is required for the activity of WRKY45 as a transcriptional activator under particular conditions. Unlike AtNPR1, OsNPR1 does not appear to be regulated by the UPS. We discuss the significance of the regulation of these rice transcriptional regulators, and compare the regulatory mechanisms between rice and Arabidopsis.

## Results

### WRKY45 protein is degraded by the ubiquitin–proteasome system

**Figure 1:**
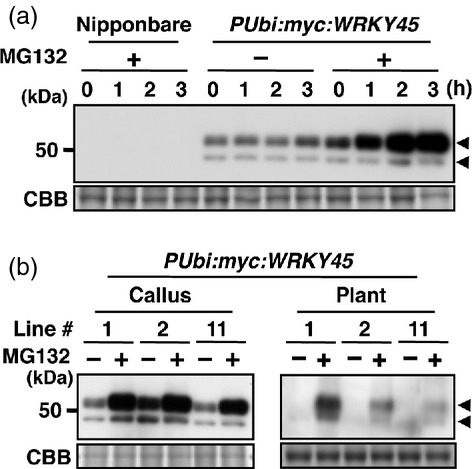
Accumulation of WRKY45 protein in rice calli and plants treated with the proteasome inhibitor MG132. (a) Wild-type and *PUbi:myc:WRKY45* transgenic calli were incubated in R2S medium containing 0.2% DMSO with (+) or without (−) 100 μm MG132 for up to 3 h, and myc:WRKY45 protein was detected using anti-myc antibody. Two or more bands were observed in this and several other experiments described below: band numbers apparently varied in different experiments due to gel conditions. Phosphatase treatment showed that the multiple bands were due to phosphorylation of WRKY45 (Figure S6). (b) Three independent lines of *PUbi*:myc:WRKY45 transgenic calli and leaf discs of plants regenerated from them were incubated with (+) or without (−) 100 μm MG132 (in 0.2% DMSO) for 6 h, and proteins were detected as in (a). Equal amounts of total proteins were loaded onto the gel. For mock treatments (without MG132), the calli and plants were incubated in R2S medium or water, respectively, containing 0.2% DMSO.

To investigate post-translational regulation of WRKY45, we constructed transgenic rice lines over-expressing myc-tagged WRKY45 driven by the constitutive maize ubiquitin promoter (*PUbi:myc:WRKY45*). These transformants were blast-resistant (see Figure 6c), indicating that the myc-tagged WRKY45 is functional. To examine the post-translational regulation of WRKY45, we treated the *PUbi:myc:WRKY45* calli with MG132, an inhibitor of the 26S proteasome, and monitored the level of myc:WRKY45 protein over time by Western blotting. As shown in Figure 1a, myc:WRKY45 protein markedly accumulated after MG132 treatment, whereas there was no significant change after mock treatment. The effect of MG132 appeared as early as 1 h after its addition. Similar results were consistently obtained in three independent lines of *PUbi:myc:WRKY45* transgenic calli (Figure 1b). Moreover, myc:WRKY45 also accumulated in MG132-treated leaf discs from *PUbi:myc:WRKY45* transgenic rice seedlings (Figure 1b). The effects of MG132 on WRKY45 protein levels were also observed when *myc:WRKY45* expression was driven by the constitutive *EF1α* promoter or a dexamethasone-inducible promoter (Figure S1). Transcript levels of *myc:WRKY45* were not affected by MG132 treatment in these transformants (Figure S2). Therefore, we conclude that the effects of MG132 on the amount of WRKY45 protein occur at the post-transcriptional level.

**Figure 2:**
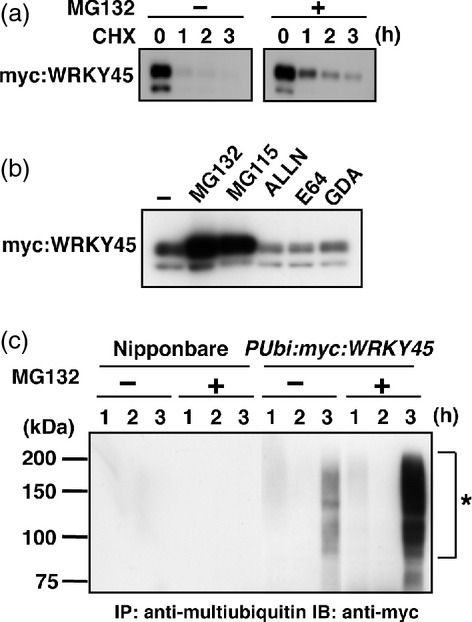
Degradation of WRKY45 by the UPS. (a) WRKY45 is a short-lived protein. *PUbi:myc:WRKY45* calli were incubated with or without 100 μm MG132 for 3 h as described in Figure 1, then the protein synthesis inhibitor cycloheximide (CHX) was added, with incubation for for additional periods. Samples were analyzed for myc:WRKY45 protein at various time points after addition of cycloheximide. (b) Proteasome inhibitors specifically stabilized WRKY45 protein. *PUbi:myc:WRKY45* calli were incubated with various proteasome or protease inhibitors for 3 h, and myc:WRKY45 protein was detected by Western blotting using anti-myc antibody. (c) Ubiquitination of WRKY45 *in vivo*. Total extracts of Nipponbare and *PUbi:myc:WRKY45* rice calli with or without MG132 treatment were subjected to immunoprecipitation using anti-multiubiquitin antibody. Polyubiquitinated myc:WRKY45 (indicated by the asterisk) was detected by Western blotting with anti-myc antibody. For mock treatments, the calli were incubated in 0.2% DMSO.

When *PUbi:myc:WRKY45* transgenic rice calli were treated with the protein synthesis inhibitor cycloheximide, myc:WRKY45 protein rapidly disappeared (half-life of <1 h), and the rate of disappearance was slowed by MG132 (Figure 2a). These results suggest that the disappearance of WRKY45 in cycloheximide-treated calli is at least partly due to 26S proteasome activity and does not require new protein synthesis. We examined the effects of several other inhibitors of protein degradation on the amount of WRKY45 protein. Under our experimental conditions, the 26S proteasome inhibitor MG115 also induced myc:WRKY45 accumulation, but the weak 26S proteasome inhibitor *N*-acetyl-Leu-Leu-norleucinal did not (Figure 2b). The cysteine protease inhibitor E-64 and the HSP90 inhibitor geldanamycin also did not affect the protein level of myc:WRKY45.

Protein degradation by the 26S proteasome is normally preceded by polyubiquitination of proteins, which serves as a marker to target them for degradation. Thus, we examined polyubiquitination of myc:WRKY45 protein in *PUbi:myc:WRKY45* rice calli. Extracts from *PUbi:myc:WRKY45* rice calli were immunoprecipitated using an anti-multiubiquitin antibody, and the precipitates were separated by SDS–PAGE. Then, the gel blot was reacted with an anti-myc antibody to visualize polyubiquitinated myc:WRKY45. As shown in Figure 2c, samples from mock-treated *PUbi:myc:WRKY45* rice calli showed a slowly migrating smeared ladder of bands, which presumably correspond to polyubiquitinated forms of myc:WRKY45. The band intensity was greatly enhanced when calli were treated with MG132. These bands were not detected in precipitants from Nipponbare rice calli. Collectively, these results indicate that WRKY45 undergoes protein degradation mediated by the UPS.

### WRKY45 degradation occurs in nuclei

**Figure 3:**
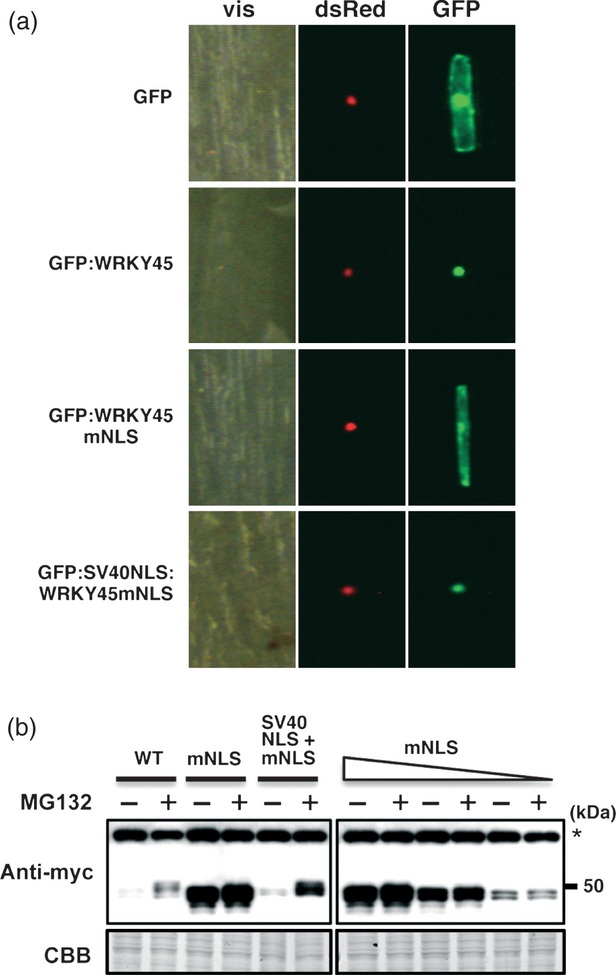
UPS-dependent degradation of WRKY45 in nuclei. (a) Subcellular localization of eGFP:WRKY45 and its nuclear localization mutants. The nuclear localization signal (NLS) of WRKY45 was disrupted by replacing two arginines in putative NLS (GGKRKAPAADRKAN) by alanines (WRKY45mNLS). SV40-derived NLS fused to the N-terminus (SV40NLS:WRKY45mNLS) restored localization to nuclei. eGFP fusion proteins with wild-type and WRKY45 derivatives were transiently expressed in rice coleoptile cells after delivery of fusion genes by particle bombardment. (b) Nuclear localization is required for UPS-dependent degradation of WRKY45. Constructs for myc-tagged WRKY45 derivatives (3 μg each) were co-delivered with *PUbi*:*myc*:*GUS* (transfection control) to rice protoplasts and incubated with (+) or without (−) MG132 for 4 h. Then, myc:WRKY45 derivatives and myc:GUS proteins (asterisk) were detected by Western blotting using anti-myc antibody. For WRKY45mNLS, decreasing amounts of plasmids (3, 1 and 0.3 μg) were also tested to rule out the possibility that the high protein levels masked the inhibition of proteasome degradation by MG132 (right panel). For mock treatments (−), the calli were incubated in 0.2% DMSO.

The subcellular localization of WRKY45 was determined using a chimeric eGFP:WRKY45 fusion protein. eGFP–WRKY45 transiently expressed in rice coleoptile cells was predominantly detected in the nuclei (Figure 3a), consistent with its function as a transcription factor. Nuclear localization of eGFP:WRKY45 was also observed when expressed in protoplasts (Figure S3). Mutations in the putative nuclear localization signal (NLS) sequence of WRKY45 resulted in cytoplasmic distribution of the fusion protein (eGFP:WRKY45mNLS, Figure 3a). Addition of the SV40 NLS to the mNLS derivative (eGFP:SV40NLS:WRKY45mNLS) restored nuclear localization. To identify the subcellular compartment in which WRKY45 is degraded by the UPS, we expressed the same WRKY45 derivatives (without GFP) in rice protoplasts and monitored the protein levels. As shown in Figure 3b, WRKY45mNLS accumulated to high levels even in the absence of MG132, and no further accumulation was observed in the presence of MG132. Moreover, addition of the SV40 NLS to WRKY45mNLS restored proteasomal degradation (Figure 3b). These results demonstrate that UPS-dependent degradation of WRKY45 occurs in the nuclei.

### Inhibition of WRKY45 degradation induces transient up-regulation of WRKY45-regulated genes

**Figure 4:**
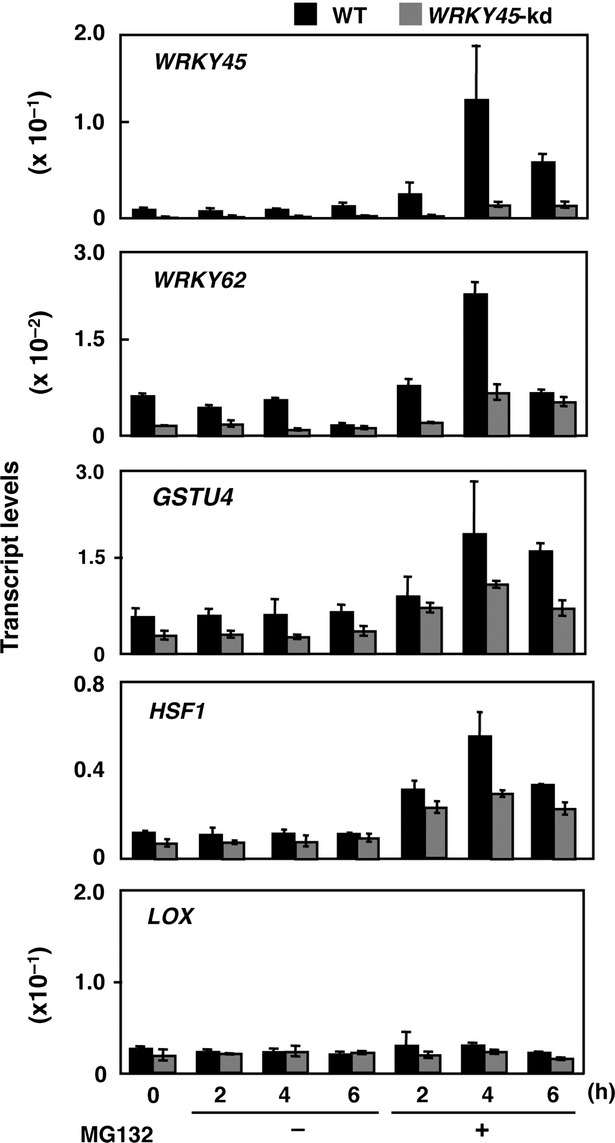
Expression of endogenous *WRKY45* and WRKY45-regulated genes in response to MG132. Discs of the 5th leaves of Nipponbare or *WRKY45* knockdown (Shimono *et al*., 2007) rice plants were incubated with or without MG132 for indicated periods, and transcript levels of *WRKY45*, *WRKY62*, *GSTU4*, *HSF1* and *LOX* genes were analyzed by quantitative RT–PCR. The transcript abundance was normalized to that of *Actin1*. Values are means ± SD of three determinations. For mock treatments (−), the leaf discs were incubated in 0.2% DMSO.

To investigate the possible involvement of proteasome-dependent degradation of WRKY45 in the transcriptional activity of WRKY45, we analyzed the expression of putative target genes of WRKY45. WRKY45 regulates the transcription of its own gene (AN, HT, unpublished results). After MG132 treatment of leaves from Nipponbare and *WRKY45* knockdown rice plants, transcripts for endogenous *WRKY45* were up-regulated as a result of auto-regulation in Nipponbare rice (Figure 4). *WRKY62* (AK067834), *GSTU4* (AK103453) and *HSF1* (AK100412) are BTH-inducible (Shimono *et al*., 2007), and their expression is WRKY45-dependent (Figure 4) (AN, HT, unpublished results). The transcript level of *WRKY62* was also up-regulated after MG132 treatment, and the up-regulation of these genes was compromised in *WRKY45* knockdown lines (Figure 4). These results suggest that accumulation of WRKY45, which resulted from inhibition of its degradation by the UPS, led to up-regulation of WRKY45-regulated genes. MG132 treatment did not affect expression of the *LOX* (AK241395) gene (Figure 4), which is SA/BTH-responsive but WRKY45-independent (Qiu *et al*., 2007). These results suggest that the effects of MG132 on the expression of WRKY45-regulated genes are due to direct inhibition of WRKY45 degradation by UPS.

### Proteasome inhibition reduces SA-induced accumulation of endogenous WRKY45

**Figure 5:**
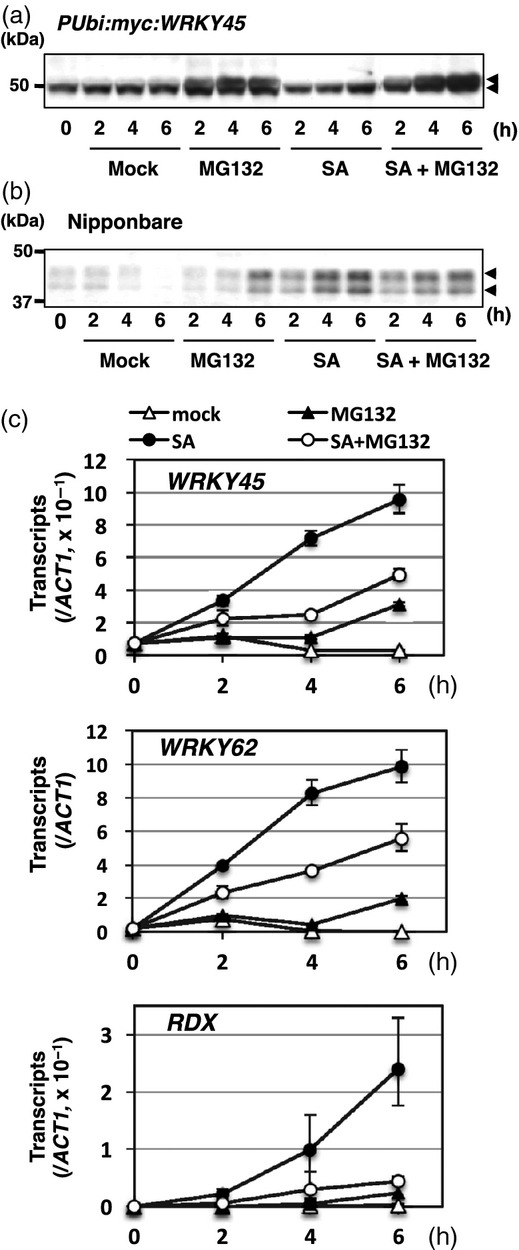
Changes in levels of WRKY45 proteins and WRKY45-regulated genes in response to SA and MG132 in Nipponbare and transformant rice plants. (a) myc:WRKY45 protein levels after SA and/or MG132 treatment in *PUbi:myc:WRKY45* transformants. Leaf disks (5th leaves) from *PUbi:myc:WRKY45* rice plants were soaked in aqueous solutions containing 1 mm SA and/or 0.1 mm MG132 for indicated periods. Total protein was analyzed by Western blotting using anti-myc antibody. The arrowheads indicate myc:WRKY45. The presence of two bands is due to phosphorylation (Figure S6). (b) Endogenous WRKY45 protein levels after SA and/or MG132 treatment in Nipponbare. Leaf disks (5th leaves) from Nipponbare were treated with 1 mm SA and/or 0.1 mm MG132, and analyzed by Western blotting using anti-WRKY45 antibody. The arrowheads indicate endogenous WRKY45. (c) Transcript levels of WRKY45-regulated genes after SA and/or MG132 treatments in Nipponbare. Transcript levels of WRKY45-regulated genes (*WRKY45*, *WRKY62* and *RDX*) were analyzed by quantitative RT–PCR (relative to *actin1*). Values are means ± SD of two determinations. We obtained similar results in three similar experiments. Aqueous solutions containing 0.2% DMSO were used for control treatments (mock and SA).

*WRKY45* transcript levels greatly increase in response to BTH and SA (Shimono *et al*., 2007). To examine whether SA influences the post-translational regulation of myc:WRKY45 protein, SA was added to leaf discs of *PUbi*:*myc:WRKY45* and the amount of myc:WRKY45 protein was monitored over time (Figure 5a). There was no significant change in myc:WRKY45 levels after addition of SA, in contrast to the increased myc:WRKY45 levels after MG132 treatment. BTH treatment also did not affect the WRKY45 level in *PUbi*:*myc:WRKY45* plants (Figure S4). These results indicate that the SA/BTH pathway does not regulate WRKY45 protein degradation.

Next, we examined the effects of MG132 on SA-induced accumulation of endogenous WRKY45 proteins in leaf discs of untransformed Nipponbare (Figure 5b). MG132 treatment increased the levels of endogenous WRKY45 proteins and SA treatment had the same effect, as expected from the SA/BTH inducibility of *WRKY45* transcription. However, we did not observe any additive effects of SA and MG132 when the leaf discs were treated with both simultaneously. Instead, unexpectedly, MG132 appeared to inhibit the SA-induced increase in the endogenous WRKY45 level. To investigate this phenomenon further, we analyzed the expression of WRKY45-regulated genes under these conditions (Figure 5c). After MG132 treatment of leaf discs of Nipponbare rice plants, transcripts for endogenous *WRKY45* were up-regulated due to auto-regulation. Those of the WRKY45-regulated genes *WRKY62* and *RDX* (AK104089, AN, HT, unpublished results) were also up-regulated after MG132 treatment. However, when rice leaves were treated with SA and MG132 simultaneously, we did not observe any additive effects of SA and MG132. Instead, MG132 treatment partially compromised SA-induced up-regulation of these genes. Collectively, these results suggest that the inhibition of WRKY45 degradation by MG132 in the presence of SA negatively affects the transcriptional activity of WRKY45. This observation raised the possibility that UPS degradation of WRKY45 is required for full activation of the transcriptional activity of WRKY45 when the SA pathway is activated.

### Deletion of a WRKY45 C-terminal region abolished both transcriptional activity and UPS-dependent degradation of WRKY45

**Figure 6:**
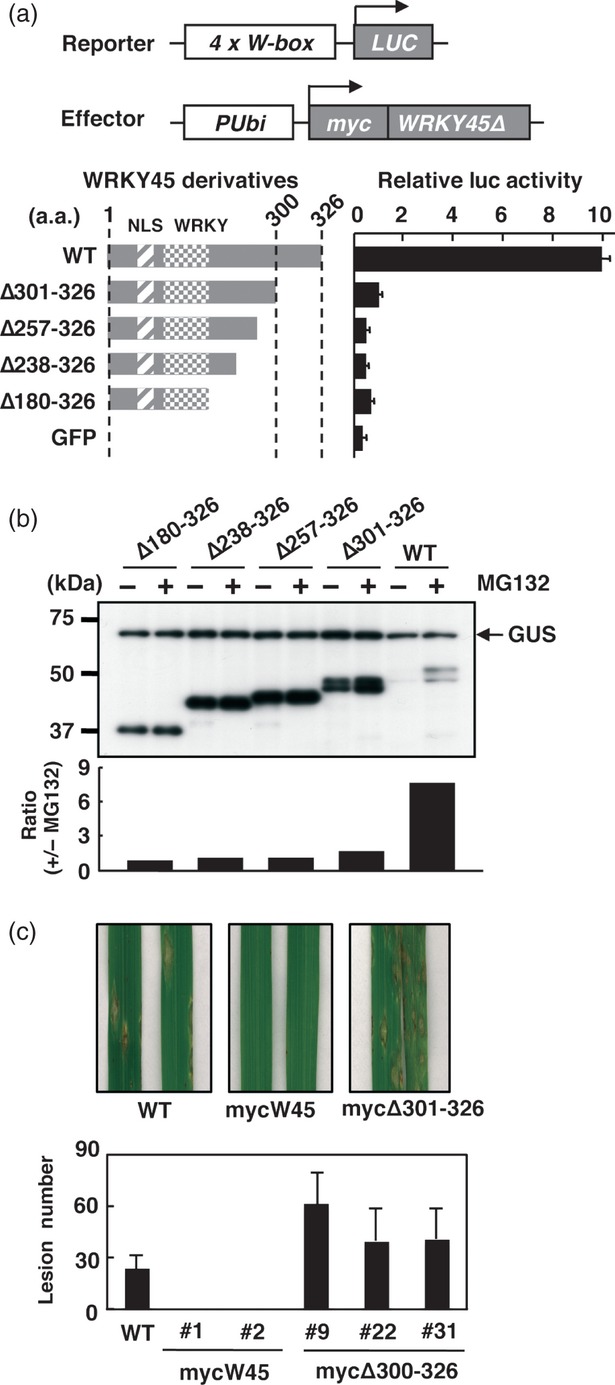
The C-terminal region is essential for transactivation activity and UPS degradation of WRKY45, and is required for induction of blast resistance. (a) Transactivation assay. The reporter construct contained four W-boxes upstream of the luciferase coding sequence. Effector constructs contained myc-tagged deletion derivatives of the WRKY45 coding sequence downstream of the maize ubiquitin promoter (*PUbi*). In WRKY45 derivatives, hatched and checked boxes represent NLS and WRKY domains, respectively. The reporter, effector and reference (*PUbi:hRLUC*) plasmids were delivered into the leaf sheath by particle bombardment. Luciferase activities were determined and normalized to the reference humanized Renilla luciferase (hRLUC) activity. The means of activities for three independent samples are represented by arbitrary units ± SD. (b) UPS degradation assay. Rice protoplasts were transfected with effector constructs for myc-tagged WRKY45 derivatives used in (a), together with a reference construct containing a *PUbi*-driven *myc:GUS* gene. Protoplasts were incubated with (+) or without (−) 50 μm MG132 for 4 h, and accumulation of each WRKY45 derivative protein was monitored by Western blotting using anti-myc antibody. Ratios of band intensities for WRKY45 derivatives in the presence or absence of MG132 are shown under the band patterns. Solutions containing 0.2% DMSO were used for mock treatments. Experiments were duplicated with similar results. Data from one representative experiment are shown. (c) Blast resistance assay. Fifth leaves of Nipponbare, *PUbi:myc:WRKY45* (mycW45) and *PUbi:myc:WRKY45Δ301–326* (mycΔ301–326) plants were spray inoculated with *Magnaporthe oryzae* conidia. Top: blast disease symptoms on 5th leaves 1 week after inoculation. Bottom: number of susceptible-type blast lesions on 5th leaves. Mean lesion numbers in 16 plants from each independent line are shown ± SD. Western blot analysis showed that expression levels of transgene-derived WRKY45 proteins in *PUbi:myc:WRKY45Δ301-326* were higher than those in *PUbi:myc:WRKY45*.

In some cases, UPS-dependent degradation of a transcription factor is required for its full activity as a transcriptional activator (Kodadek *et al*., 2006). To investigate the relationship between transcriptional activity and UPS-dependent degradation of WRKY45, we generated a deletion series of the WRKY45 coding sequence and examined their transcriptional activity and protein degradation. The amino acid 257–300 and 262–326 regions of WRKY45, when fused with the GAL4 DNA binding domain, showed transactivation activity in yeast (Figure S5); therefore, we focused on this region (257–326) for further deletion analyses. For transactivation assays, we used microprojectiles to co-deliver effector constructs containing truncated forms of WRKY45 and a reporter construct containing W-box sequences fused upstream of the luciferase reporter gene (Shimono et al., 2007) into rice coleoptiles. The results showed that a C-terminal deletion of 26 amino acids (amino acids 301–326) abolished most of the transactivation activity of WRKY45 (Figure 6a, D301–326).

Next, we investigated the relationship between the domains required for transcriptional activity and UPS degradation of WRKY45. We performed protein degradation assays for the C-terminal deletion series described above using transient expression assays in cultured rice cells. The results showed that the C-terminal deletion of 26 amino acids (Δ301–326) reduced protein degradation to one-fifth of that observed with the full-length WRKY45 (Figure 6b). A further deletion (Δ258–326) reduced protein degradation only slightly further, and additional deletions had no effect. Thus, the C-terminal 26 amino acid region was found to be essential for UPS-dependent degradation of WRKY45, as well as for transcriptional activity of WRKY45. These results suggest that there is a link between UPS-dependent degradation and transcriptional activity of WRKY45.

To further examine the functional relevance of the WRKY45 C-terminus region, we generated transgenic rice plants constitutively over-expressing myc:WRKY45Δ301–326, and examined their blast disease symptoms 7 days after fungal inoculation. In contrast to myc:WRKY45-over-expressing rice plants, which exhibited strong blast resistance, myc:WRKY45Δ301-326 over-expressing rice plants were not resistant, and, in fact, were slightly more susceptible to blast disease than Nipponbare (Figure 6c). Therefore, the short C-terminal peptide is essential for the function of WRKY45 to activate resistance to blast disease, although this does not demonstrate the relevance of the proteasome degradation *per se* for activating the disease resistance.

### Transactivation activity and UPS degradation of WRKY45 are separable

**Figure 7:**
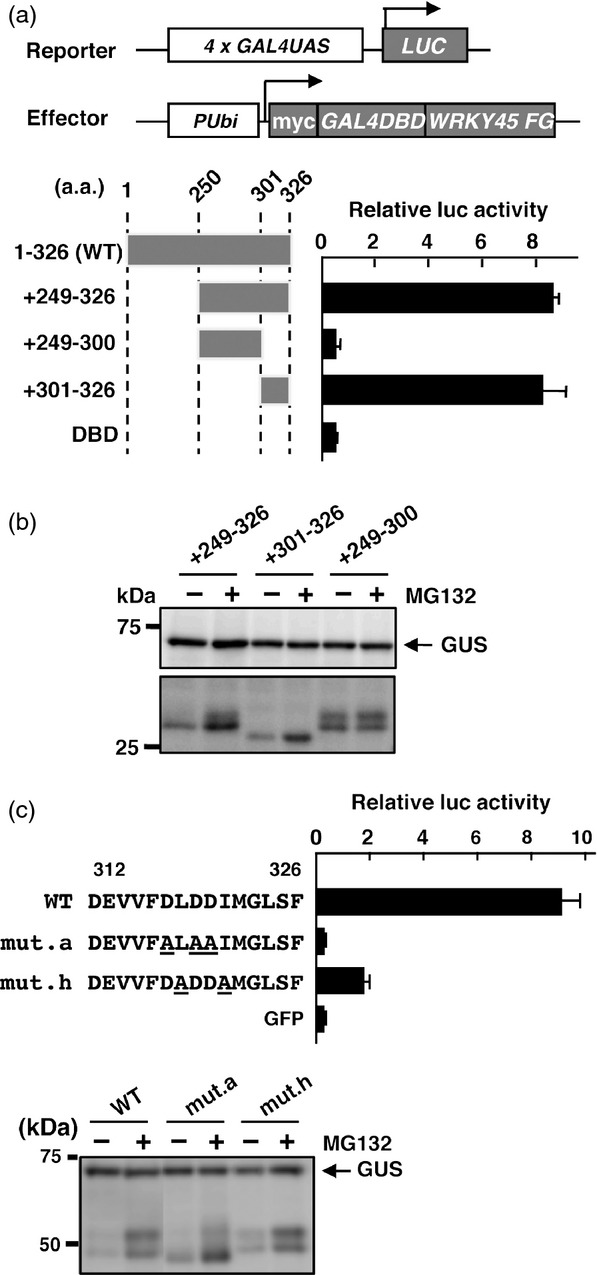
The C-terminal region of WRKY45 is sufficient for transactivation activity and UPS-dependent degradation. (a) Transactivation assay. The reporter construct contained four copies of the Upstream activation sequence (GAL4UAS), which is the GAL4 binding sequence, upstream of the luciferase coding sequence. Effector constructs contained a *myc:tagged* GAL4 DNA-binding domain (GAL4DBD) fused to partial fragments of *WRKY45* (*WRKY45 FG*) at the C-terminus under the control of the *PUbi* promoter. Transactivation assays were performed as described in Figure 6(a). (b) UPS degradation assay. Rice protoplasts were transfected with reporter constructs containing myc:GAL4DBD-fused *WRKY45* partial fragments as used in (a) under the control of the *PUbi* promoter together with the reference *myc*:*GUS* construct, and analyzed as described in Figure 6(b). Upper and lower parts of the same gel are shown, with size markers indicated. (c) Mutational analysis of the C-terminus of WRKY45. mut.a and mut.h mutants contained alanine substitutions (underlined) of acidic or hydrophobic amino acids within the C-terminal region of WRKY45, respectively. Transactivation assays (upper) and degradation assays (lower) were performed as described in (a) and (b), respectively. DMSO solutions (0.2%) were used for mock treatments in (b) and (c).

To further investigate the relationship between the transactivation activity and UPS degradation of WRKY45, we examined whether addition of the C-terminus peptide of WRKY45 influences transactivation activity and protein stability in heterologous systems. In a transactivation assay in cultured rice cells (Figure 7a), addition of the C-terminal portion (amino acids 249–326) of WRKY45 to the GAL4 DNA binding domain (GAL4DBD) resulted in strong transactivation of a reporter gene containing the GAL4 binding sequence (UAS) upstream of the luciferase gene (Figure 7a, +249–326). Deletion of amino acids 301–326 from this region eliminated the transactivation activity almost completely (Figure 7a, +249–300). Addition of the 301–326 region to GAL4DBD conferred strong transactivation activity to a level comparable with that of the 249–326 region, but addition of the 249–300 region did not. In a UPS degradation assay in cultured rice cells, GAL4DBD fused to the 301–326 region or the 249–326 region was susceptible to UPS degradation, whereas GAL4DBD fused to the 249–300 region was not (Figure 7b). These results showed that the C-terminal 301–326 peptide of WRKY45 is sufficient for both transactivation and UPS degradation.

The 301–326 peptide includes a short stretch of amino acids (DLDDI) consisting of acidic amino acids and hydrophobic amino acids, which is somewhat similar to the nine amino acid transactivation domain, a motif that is conserved in activation domains of animal, yeast and viral transcription factors (Piskacek *et al*., 2007). Based on this finding, we introduced alanine substitutions in place of either acidic or hydrophobic amino acids in the DLDDI motif of WRKY45 (Figure 7c). In a transactivation assay, both types of mutations disrupted the transactivation activity of WRKY45 (Figure 7c), indicating that this region does indeed constitute an activation domain. However, MG132-sensitive degradation of these mutants was comparable to that of wild-type WRKY45 (Figure 7c). These results indicate that the amino acid residues required for transactivation activity are not required for UPS degradation of WRKY45; thus, the regions essential for the two activities of WRKY45 are separable at the amino acid level. We also mutated lysine at position 310, which is the only candidate site for ubiquitination in the 301–326 region; however, this mutation did not affect WRKY45 degradation or transactivation (data not shown). This suggests that a possible modification in the 301–326 region regulates ubiquitination at the site upstream of this region.

### OsNPR1 is not regulated by the UPS

**Figure 8:**
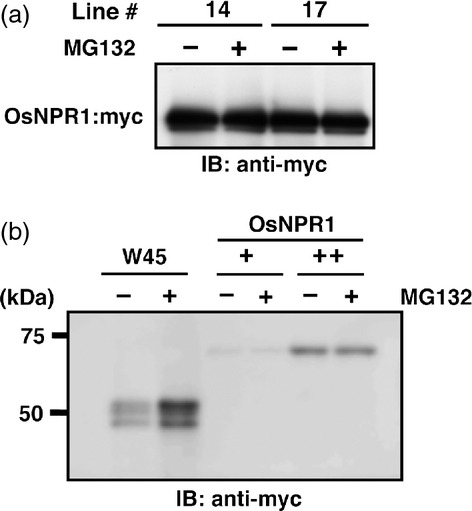
OsNPR1 is not regulated by the UPS. (a) Leaf disc assay. Leaf discs (5th leaves) from *PUbi:OsNPR1:myc* transgenic rice plants were incubated with or without 0.1 mm MG132 for 6 h. Total proteins were analyzed by Western blotting using anti-myc antibody. (b) Transient expression assay. Rice protoplasts were transfected with *PUbi:myc:WRKY45* (10 μg) or *PUbi:OsNPR1:myc* DNA [0.01 μg (+) or 0.1 μg (++)]. The protoplasts were treated with solutions containing 100 μm MG132 (+) or solvent (0.2% DMSO) only (−) for 4 h before lysis.

We previously proposed that the SA pathway in rice branches into WRKY45-dependent and OsNPR1-dependent sub-pathways (Shimono *et al*., 2007), and the two regulators transcriptionally largely regulate different target genes (Sugano *et al*., 2010). The finding of UPS-dependent regulation of AtNPR1 (Spoel *et al*., 2009) prompted us to investigate whether regulation of OsNPR1 is also regulated by UPS in rice. We treated leaf discs from *Ubi:OsNPR1:myc* transgenic rice plants with MG132 and determined the level of OsNPR1:myc protein, which we showed to be functional (Sugano *et al*., 2010). However, unlike AtNPR1, the amount of OsNPR1 protein did not change after MG132 treatment (Figure 8a). We also examined the effects of MG132 on the amounts of myc:WRKY45 and OsNPR1:myc proteins transiently expressed in rice protoplasts. As shown in Figure 8b, the level of OsNPR1 protein was not affected by addition of MG132, whereas MG132 markedly stabilized myc:WRKY45. These results suggest that OsNPR1 is not regulated by the UPS in rice, at least under uninfected conditions, unlike its counterpart in Arabidopsis.

## Discussion

During defense responses in plants, substantial amounts of energy and resources that are otherwise dedicated to growth and development are allocated to activate a body of defense genes at the cost of plant growth. In addition, the defense reactions themselves may directly damage cellular activities in plants. Therefore, the defense systems in plants must be tightly regulated to avoid their untimely activation. UPS-dependent protein degradation has emerged as an important survival tactic adopted by both plants and microbes during their interactions (Dielen *et al*., 2010). Spoel *et al*. (2009) reported that AtNPR1, the central regulator of the SA signaling pathway in Arabidopsis, is regulated by UPS-dependent turnover, and proposed that it plays a dual role in regulation of NPR1-mediated defense reactions. In the present study, we demonstrated that WRKY45, one of the central regulators of the SA/BTH signaling pathway in rice, is also regulated by UPS-dependent degradation, whereas OsNPR1, the rice counterpart of AtNPR1, is not.

UPS-dependent regulation of transcription factors has been extensively studied in various systems, and several biological roles have been proposed for this regulation. One simple and well-recognized role is negative regulation of the amounts of target proteins. In plants, UPS-dependent regulation is thought to be involved in diverse aspects of plant development and plant stress responses, and the amounts of transcription factors such as EIN3, Aux/IAAs and ABI3 are negatively regulated by the UPS in response to hormonal signals (Vierstra, 2009). In addition to its negative regulatory role, a positive role directly contributing to the transcriptional activity of transcription factors has been proposed in yeast and mammals (Lipford and Deshaies, 2003; Muratani and Tansey, 2003). Several studies have shown that UPS-dependent turnover of transcription factors occurs after they bind to their target promoters (Muratani and Tansey, 2003). It is proposed that recruitment of the 26S proteasome to the transcriptional initiation complex on the promoter leads to destruction of the transcription factors, which in turn converts the RNA polymerase from an initiation-competent form to an elongation-competent form. This may culminate in either full activation of transcription, strict control of the transcriptional activity of each transcription factor molecule, and/or limitation of the duration that the transcription factors work (Mavrakis *et al*., 2007). In the case of human estrogen receptor a (hERα), for example, UPS-dependent turnover is proposed to be required to clear hERα molecules from promoters after transcriptional activation. This process (refreshment of promoters) is critical for subsequent rounds of hERα-dependent transcriptional activation; thus, the turnover enhances overall transcription by hERα (Reid *et al*., 2003). In transcription factors that undergo this type of regulation by the UPS (Muratani and Tansey, 2003), the domains required for UPS-dependent degradation overlap with those required for transcriptional activity (activation domain) in many cases. In WRKY45, the two domains are also closely located, although they are distinguishable at the amino acid level. Therefore, it is likely that the mechanism proposed in mammals and yeast also holds for proteasome-dependent enhancement of the transcriptional activity of WRKY45.

**Figure 9:**
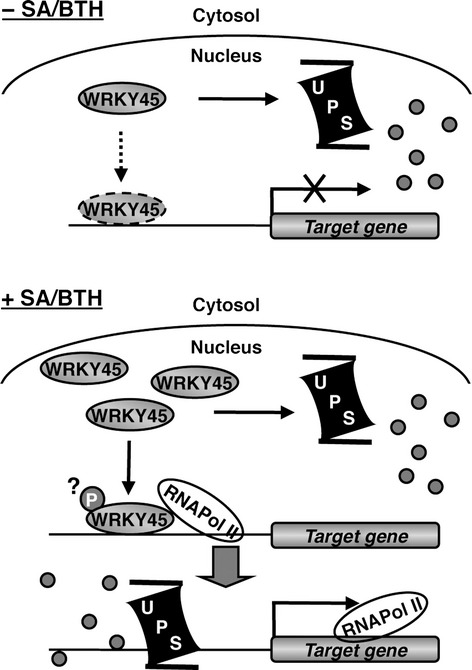
Proposed model for dual roles of UPS-dependent regulation of WRKY45. Constitutive protein degradation by the nuclear UPS eliminates WRKY45 before it reaches the promoters of target genes. This regulation most likely prevents spurious defense activation by WRKY45 in the absence of pathogen infection. Upon BTH treatment or pathogen infection, WRKY45 accumulation exceeds degradation, allowing WRKY45 to bind to target promoters. The nuclear UPS directly contributes to WRKY45-dependent transcriptional activation of target genes when the SA pathway is activated.

We propose that UPS-dependent turnover may have two contrasting roles in regulating WRKY45; negative regulation by decreasing the amount of WRKY45 protein, and a positive role directly contributing to transcriptional activity (Figure 9). The negative regulation is supported by the following observations: treatment of leaf discs with proteasome inhibitor alone increased the level of endogenous WRKY45 protein, and transcript levels of WRKY45 target genes were elevated accordingly. Thus, the UPS negatively regulates the abundance of the WRKY45 protein, which is directly correlated with the transcript levels of its target genes. As transcription of *WRKY45* may be activated by spurious signals in the absence of pathogen infection, there is a potential risk of inducing untimely defense responses that adversely affect plant growth. Indeed, our *WRKY45*-over-expressing rice transformants showed growth retardation in a growth condition-dependent manner (Shimono *et al*., 2007). However, such a risk is avoided if the UPS continuously breaks down WRKY45 in the absence of pathogens, thereby preventing costly defense responses.

Our proposal regarding a direct contribution of WRKY45 degradation to the activity of WRKY45 as a transcriptional activator is based on the following observations: SA-induced accumulation of WRKY45, which is in part due to auto-regulation of *WRKY45* transcription, was suppressed by the proteasome inhibitor. Transcriptional induction of other WRKY45-regulated genes by SA was also suppressed by simultaneous treatment with MG132. These observations cannot be explained by the well-known action of proteasome inhibitors that results in protein accumulation. The close association between the transcriptional activation domain and the domain required for UPS-dependent turnover at the C-terminal region of WRKY45, as demonstrated by loss- and gain-of-function experiments (Figures 6 and 7), provides further evidence for the existence of the direct contribution. These domains commonly overlap in transcription factors whose transcriptional activity is regulated by the UPS (Muratani and Tansey, 2003). Our further analysis of the C-terminal activation domain determined the amino acids essential for transactivation activity; these amino acids were not involved in UPS degradation. Similar results have been reported for hERa, in which transactivation activity and UPS degradation of hERa are closely associated but distinguishable at the amino acid level (Valley *et al*., 2005). However, some additional evidence, including identification of a WRKY45 mutant that is defective in turnover but retains transactivation abilities, is necessary to prove the direct contribution of proteasome turnover to transcriptional activity. At present, it remains possible that turnover of an as yet unidentified factor is necessary for SA-induced WRKY45-dependent gene activation.

UPS-mediated regulation of WRKY45 appears to enable elaborate regulation of defense responses (Figure 9). In the absence of pathogen infection, WRKY45 is present at a basal level (which occasionally increases because of spurious signals); the proteins are translocated to the nucleus but are immediately degraded via UPS-dependent turnover before they can access the promoters of target genes. Once the amount of WRKY45 protein reaches a certain level after activation of the SA/BTH signaling pathway as a result of pathogen challenge or exogenous application of chemical inducers, the accumulation of WRKY45 protein exceeds its basal turnover. Then, the surplus WRKY45 protein molecules are recruited to target promoters, where the transcriptional activity of WRKY45 is presumably enhanced by UPS-mediated turnover. The turnover may also play a role in the eventual removal of used WRKY45 proteins from the promoters, thereby preventing their re-use, which may lead to unnecessary continuation of the defense response. Thus, the UPS may also function as a built-in terminator of WRKY45-mediated SA signaling after the threat of the pathogen has subsided.

Spoel *et al*. (2009) proposed a similar dual model for the role of AtNPR1 turnover in Arabidopsis. In the absence of pathogen infection, AtNPR1 is trapped in the cytosol by *S*-nitrosylation-mediated oligomerization (Tada *et al*., 2008). A small proportion of AtNPR1 localizes to the nuclei, but it is degraded by constitutive UPS activity, thereby preventing the untimely activation of defense responses. On the other hand, the UPS positively regulates AtNPR1 activity in response to SA. Activation of the SA signaling cascade by pathogen infection alters the intracellular redox state, which facilitates monomerization of AtNPR1 and its subsequent localization to the nuclei. SA also induces phosphorylation of AtNPR1 in the nuclei and the phosphorylated AtNPR1 interacts with the E3 ligase complex containing CUL3, then undergoes UPS-dependent turnover. Both mutations in CUL3 and disruption of phosphorylation of AtNPR1 stabilized AtNPR1, but decreased expression of its target genes. Based on these observations, it was proposed that UPS-dependent turnover of AtNPR1 is required for its full activity as a transcriptional co-activator in response to SA.

Here, we showed that, in rice, OsNPR1 barely undergoes UPS-dependent degradation, at least under uninfected conditions, in contrast to its counterpart in Arabidopsis. These results may reflect differences in the roles of these transcriptional co-activators in SA/BTH signaling pathways between rice and Arabidopsis. In Arabidopsis, almost all (>99%) BTH-responsive genes are regulated by AtNPR1 (Wang *et al*., 2006). Comparable numbers of up- and down-regulated genes are AtNPR1-dependent, and the up-regulated genes include many defense-related genes, such as *PR* genes. In rice, we have previously proposed that SA/BTH-induced resistance is controlled by two regulators, WRKY45 (Shimono *et al*., 2007) and OsNPR1/NH1 (Sugano *et al*., 2010), which function in different branches of the SA/BTH pathway. Transcript profiling analysis revealed that two-thirds of BTH-responsive genes in the OsNPR1-dependent sub-pathway are down-regulated by BTH. These down-regulated genes include several genes involved in photosynthesis and protein synthesis, indicating that one of the major roles of OsNPR1 is to relocate energy and resources from housekeeping cellular activities to defense activities (Sugano *et al*., 2010). Meanwhile, almost 85% of WRKY45-dependent genes were up-regulated genes by BTH, and many are directly associated with defense, e.g. *PR* and *R* genes (AN, HT, unpublished results). Taken together, these results suggest that a major part of direct defense responses, such as induction of *PR* and *R* genes, is regulated by AtNPR1 in Arabidopsis and by WRKY45 in rice. The SA pathway in Arabidopsis is also partially NPR1-independent in early phases of activation, and WRKY70 is involved in the NPR1-independent pathway (Li *et al*., 2004; Knoth *et al*., 2007); however, the separation of the pathway appears to be more prominent in rice than in Arabidopsis. Such differences are reflected in the different patterns of UPS-dependent regulation of the major transcriptional regulators in the SA pathway under uninfected conditions. In Arabidopsis, AtNPR1 is phosphorylated upon pathogen challenge, and this leads to proteasomal degradation and full activation of AtNPR1 (Spoel *et al*., 2009). UPS degradation of OsNPR1 under pathogen challenge remains to be investigated.

To further investigate regulation of the UPS-dependent turnover of WRKY45, it is important to identify the regulatory components of the ubiquitination machinery. Turnover of WRKY45 coupled with transcriptional activation is likely to occur on promoters, whereas constitutive basal turnover of WRKY45 may occur when WRKY45 is not associated with promoters. It is possible that the two types of WRKY45 turnover require different E3 ligase complexes. Identification of such proteins will be useful in understanding the mechanisms of nuclear UPS-dependent transcriptional regulation in plant immunity.

## Experimental procedures

### Plasmid construction

To construct *PUbi:myc:WRKY45* and *PUbi:myc:WRKY45Δ301–326*, a fragment containing the *WRKY45* 5' UTR and 3 × *myc* sequences was digested using *Kpn*I and *Sfi*I at both ends, and inserted into corresponding sites in the plasmid pUCAP–KS (van Engelen *et al*., 1995). In this plasmid, the positions of *Sfi*I and *Kpn*I sites were exchanged by inserting a double-stranded linker in pUCAP/Ubi–NT (Shimono *et al*., 2007). Then, a fragment containing the 3′ UTR of WRKY45 was digested with *Bam*HI and *Not*I and inserted into the corresponding sites. Subsequently, the *WRKY45* coding sequence with or without the amino acid 301–326 region was inserted between the *Sfi*I and *Bam*HI sites. Binary vectors for plant transformation with these chimeric genes were constructed essentially as described previously (Shimono *et al*., 2007).

To construct effector plasmids for transient assays, the pUCAP–KS vector was cut with *Kpn*I and *Sfi*I, and a double-stranded 3 × *myc* linker was inserted into this site to generate pUCAP–*myc*. DNA fragments for *WRKY45* derivatives were inserted between the *Sfi*I and *Bam*HI sites of pUCAP–*myc*.

To construct *PUbi:myc:eGFP:WRKY45* and *PUbi:myc:eGFP:WRKY45mNLS*, a DNA fragment for eGFP was inserted into the *Sfi*I sites of *PUbi:myc:WRKY45* and *PUbi:myc:WRKY45mNLS*, respectively. *PUbi:myc:eGFP:SV40NLS:WRKY45mNLS* was generated by inserting an SV40 NLS linker upstream of the *WRKY45mNLS* sequence in *PUbi:myc:eGFP:WRKY45mNLS*. To construct *PUbi:OsNPR1:myc*, a DNA fragment for the OsNPR1 coding sequence lacking a termination codon was inserted into the *Sfi*I and *Bam*HI sites of pUCAP/Ubi–NT. Subsequently, a 3 × *myc* linker containing a termination codon was inserted between the *Bam*HI and *Sac*I sites. All the constructs used were confirmed by DNA sequencing.

#### Plant material

Rice plants (*Oryza sativa* cv. Nipponbare) were grown in a greenhouse at 28°C/26°C (day/night) and 60% humidity, unless otherwise noted. Rice calli were maintained on N6D medium (Toki *et al*. 2006) containing antibiotics in a growth chamber under the following conditions: 28°C/26°C (light/dark), 50% humidity, 200 mmol m^−2^ sec^−1^ light intensity, and a 14 h light/10 h dark photoperiod.

#### Blast resistance test

To test for blast resistance, conidia of blast fungus (*Magnaporthe oryzae*, race 007.0) were suspended in 0.01% Silwet L-77 (OSI Specialties Inc., Danbury, CT, USA) at a density of 10^5^ cells ml^−1^, and sprayed onto rice leaves as described previously (Shimono *et al*., 2007).

#### Inhibitor experiments

Rice calli were incubated in R2S medium (Ohira *et al*., 1973) supplemented with various inhibitors at the indicated concentrations at 28°C. For *in planta* assays, young leaves were cut into pieces and submerged in solutions containing 0.01% Silwet–L77 and 100 μm MG132. Samples were infiltrated at 0.06 MPa for 15 min, then incubated on a rotary shaker at 28°C. The samples were harvested at the indicated times, and frozen quickly in liquid nitrogen. Stock solutions of 50 mm MG132 or other inhibitors in dimethylsulfoxide (DMSO) were used for all inhibitor experiments.

#### Protein extraction and immunoblotting

To extract total proteins, frozen samples were ground into powder and suspended in SDS/urea buffer (8 M urea, 10% SDS, 20 mm Tris/HCl pH 6.8) or ice-cold TNT buffer [50 mm Tris/HCl pH 7.5, 150 mm NaCl, 0.1% Triton X–100 and one Complete EDTA-free protease inhibitor cocktail tablet (Roche, http://www.roche.com)]. The lysate was vortexed briefly and incubated on ice for 30 min. After centrifugation at 15 000 ***g*** at 4°C for 15 min, supernatants were recovered as total protein fractions. Protein concentrations in the fractions were measured by the Bradford method (Bradford, 1976). Samples with equal amounts of proteins were separated by 10% SDS–PAGE gel [total gel concentration (T): percentage cross-linking (C) = 29:1]. In some experiments, the T:C ratio was modified to improve separation of modified forms of WRKY45 proteins. Western blotting was performed using the SNAP id system (Millipore http://www.millipore.com). Briefly, proteins were transferred onto an Immune-Blot poly(vinylidene difluoride) membrane (Bio–Rad http://www.bio/rad.com) and blots were blocked using Blocking One (Nacalai https://www.nacalai.co.jp). Myc-tagged proteins were detected by reacting blots with mouse monoclonal anti-myc 9B11 (Cell Signaling http://www.cellsignal.com) or custom made rabbit polyclonal anti-WRKY45 (Scrum, http://www.scrum-net.co.jp), then horseradish peroxidase-conjugated secondary antibody (Jackson ImmunoResearch http://www.jacksonimmuno.com). Bands were visualized using ECL plus (GE Healthcare http://www.gehealthcare.com) or Luminata Forte (Millipore) detection systems.

#### Immunoprecipitation with anti-multiubiquitin antibody

Transgenic rice calli were ground in liquid nitrogen, and the resulting powder was suspended in three volumes of 50 mm Tris/HCl pH7.5, 150 mm NaCl, 0.05% Nonidet P-40, one Complete EDTA-free protease inhibitor cocktail tablet (Roche) and 50 μm MG132. After centrifugation at 15 000 ***g*** for 10 min at 4°C, supernatants were recovered and supplied with 20 μl of anti-multiubiquitin antibody (FK2, MBL http://www.mbl.co.jp) per 1 mg protein, rotated for 2 h at 4°C, and centrifuged at 15 000 ***g*** for 10 min at 4°C. The pellets were washed briefly 3 times with the buffer, separated by 10% SDS–PAGE, and analyzed by Western blotting using anti-myc antibody as described above.

#### Detection of eGFP fluorescence

Localization of eGFP fusion proteins was visualized by GFP fluorescence under UV light and photographed under a Leica DMR microscope (http://www.leica-camera.com/).

#### Quantitative PCR analysis

Reverse transcription was performed using 2 μg total RNA treated with DNase I (Invitrogen http://www.invitrogen.jp/) using SuperScript III (Invitrogen) and oligo(dT)_23_ primers (Sigma-Aldrich, http://www.sigmaaldrich.com). Quantitative RT–PCR was performed on a Thermal Cycler Dice TP800 system (Takara Bio, http://www.takara-bio.co.jp) using SYBR premix Ex Taq II mixture (Takara Bio) with cycles of 95°C for 5 sec and 60°C for 30 sec. Rice *Actin1* (AK100267) was used as an internal standard. Primers for PCR used in this study are listed in Table S1. These primer sets were tested by dissociation curve analyses and verified for the absence of non-specific amplification.

#### Transient expression assay

For transactivation assays, inner leaf sheaths of rice were cut into pieces and placed side by side on agar plates containing 0.4 M mannitol. Effector (1.5 μg), reporter (2.0 μg) and reference (0.7 μg) plasmids were introduced into the leaf sheaths using a PDS–1000/He biolistic particle delivery system (Bio–Rad). After incubation at 28°C overnight, samples were collected and ground in liquid nitrogen. Luciferase activities were assayed using a DualGlo luciferase reporter assay system (Promega, http://www.promega.com). To investigate the stability of WRKY45, plasmids were transfected into protoplasts of rice Oc cells by electroporation as described previously (Kawakatsu *et al*., 2009). The band intensity of WRKY45 proteins in Western blots was measured using ImageJ software (http://rsb.info.nih.gov/ij/).

## SUPPORTING INFORMATION

**Figure S1.** Accumulation of WRKY45 protein under proteasome inhibition.

**Figure S2.** The transcript level of *myc:WRKY45* is unchanged by proteasome inhibition.

**Figure S3.** Localization of the WRKY45:GFP fusion protein in the nucleus.

**Figure S4.** Effect of BTH treatment on the protein level of myc: WRKY45.

**Figure S5.** The transactivation domain of WRKY45 is located at the C-terminus.

**Figure S6.** WRKY45 is phosphorylated.

**Table S1.** Primers used for quantitative PCR.

## References

[b101] Bradford M (1976). A rapid and sensitive for the quantitation of microgram quantitites of protein utilizing the principle of protein-dye binding. Anal. Biochem.

[b1] Chern M, Fitzgerald HA, Canlas PE, Navarre DA, Ronald PC (2005). Overexpression of a rice NPR1 homolog leads to constitutive activation of defense response and hypersensitivity to light. Mol. Plant Microbe Interact.

[b2] Delaney TP, Friedrich L, Ryals JA (1995). Arabidopsis signal transduction mutant defective in chemically and biologically induced disease resistance. Proc. Natl Acad. Sci. USA.

[b3] Despres C, DeLong C, Glaze S, Liu E, Fobert PR (2000). The Arabidopsis NPR1/NIM1 protein enhances the DNA binding activity of a subgroup of the TGA family of bZIP transcription factors. Plant Cell.

[b4] Dielen AS, Badaoui S, Candresse T, German-Retana S (2010). The ubiquitin/26S proteasome system in plant–pathogen interactions: a never-ending hide-and-seek game. Mol. Plant Pathol.

[b5] Durrant WE, Dong X (2004). Systemic acquired resistance. Annu. Rev. Phytopathol.

[b104] van Engelen FA, Molthoff JW, Conner AJ, Nap JP, Pereira A, Stiekema WJ (1995). PBINPLUS : an improved plant transformation vector based on pBIN19. Transgenic Res.

[b6] Eulgem T, Somssich IE (2007). Networks of WRKY transcription factors in defense signaling. Curr. Opin. Plant Biol.

[b7] Eulgem T, Rushton PJ, Schmelzer E, Hahlbrock K, Somssich IE (1999). Early nuclear events in plant defence signalling: rapid gene activation by WRKY transcription factors. EMBO J.

[b8] Eulgem T, Rushton PJ, Robatzek S, Somssich IE (2000). The WRKY superfamily of plant transcription factors. Trends Plant Sci.

[b9] Heidel AJ, Dong X (2006). Fitness benefits of systemic acquired resistance during *Hyaloperonospora parasitica* infection in *Arabidopsis thaliana*. Genetics.

[b10] Heidel AJ, Clarke JD, Antonovics J, Dong X (2004). Fitness costs of mutations affecting the systemic acquired resistance pathway in *Arabidopsis thaliana*. Genetics.

[b11] Heil M, Baldwin IT (2002). Fitness costs of induced resistance: emerging experimental support for a slippery concept. Trends Plant Sci.

[b12] Iwai T, Seo S, Mitsuhara I, Ohashi Y (2007). Probenazole-induced accumulation of salicylic acid confers resistance to *Magnaporthe grisea* in adult rice plants. Plant Cell Physiol.

[b13] Kalde M, Barth M, Somssich IE, Lippok B (2003). Members of the Arabidopsis WRKY group III transcription factors are part of different plant defense signaling pathways. Mol. Plant Microbe Interact.

[b14] Kawakatsu T, Yamamoto MP, Touno SM, Yasuda H, Takaiwa F (2009). Compensation and interaction between RISBZ1 and RPBF during grain filling in rice. Plant J.

[b15] Kinkema M, Fan W, Dong X (2000). Nuclear localization of NPR1 is required for activation of *PR* gene expression. Plant Cell.

[b16] Knoth C, Ringler J, Dangl JL, Eulgem T (2007). Arabidopsis WRKY70 is required for full RPP4-mediated disease resistance and basal defense against *Hyaloperonospora parasitica*. Mol. Plant Microbe Interact.

[b17] Kodadek T, Sikder D, Nalley K (2006). Keeping transcriptional activators under control. Cell.

[b18] Li J, Brader G, Palva ET (2004). The WRKY70 transcription factor: a node of convergence for jasmonate-mediated and salicylate-mediated signals in plant defense. Plant Cell.

[b19] Lipford JR, Deshaies RJ (2003). Diverse roles for ubiquitin-dependent proteolysis in transcriptional activation. Nat. Cell Biol.

[b20] Loake G, Grant M (2007). Salicylic acid in plant defence – the players and protagonists. Curr. Opin. Plant Biol.

[b21] Luo M, Dennis ES, Berger F, Peacock WJ, Chaudhury A (2005). MINISEED3 (MINI3), a WRKY family gene, and *HAIKU2**IKU2*), a leucine-rich repeat (LRR) KINASE gene, are regulators of seed size in Arabidopsis. Proc. Natl Acad. Sci. USA.

[b22] Mavrakis KJ, Andrew RL, Lee KL, Petropoulou C, Dixon JE, Navaratnam N, Norris DP, Episkopou V (2007). Arkadia enhances nodal/TGF–β signaling by coupling phospho-Smad2/3 activity and turnover. PLoS Biol.

[b23] Miao Y, Laun T, Zimmermann P, Zentgraf U (2004). Targets of the WRKY53 transcription factor and its role during leaf senescence in Arabidopsis. Plant Mol. Biol.

[b24] Mou Z, Fan W, Dong X (2003). Inducers of plant systemic acquired resistance regulate NPR1 function through redox changes. Cell.

[b25] Muratani M, Tansey WP (2003). How the ubiquitin–proteasome system controls transcription. Nat. Rev. Mol. Cell Biol.

[b201] Ohira K, Ojima K, Fujiwara A (1973). Studies on nutrition of rice cell-culture.1. Simple, defined medium for rapid growth in suspension culture. Plant Cell Physiol.

[b26] Piskacek S, Gregor M, Nemethova M, Grabner M, Kovarik P, Piskacek M (2007). Nine-amino-acid transactivation domain: establishment and prediction utilities. Genomics.

[b27] Qiu D, Xiao J, Ding X, Xiong M, Cai M, Cao Y, Li X, Xu C, Wang S (2007). OsWRKY13 mediates rice disease resistance by regulating defense-related genes in salicylate- and jasmonate-dependent signaling. Mol. Plant Microbe Interact.

[b28] Reid G, Hubner MR, Metivier R, Brand H, Denger S, Manu D, Beaudouin J, Ellenberg J, Gannon F (2003). Cyclic, proteasome-mediated turnover of unliganded and liganded ERα on responsive promoters is an integral feature of estrogen signaling. Mol. Cell.

[b29] Ren X, Chen Z, Liu Y, Zhang H, Zhang M, Liu Q, Hong X, Zhu J–K, Gong Z (2010). ABO3, a WRKY transcription factor, mediates plant responses to abscisic acid and drought tolerance in Arabidopsis. Plant J.

[b30] Shimono M, Sugano S, Nakayama A, Jiang CJ, Ono K, Toki S, Takatsuji H (2007). Rice WRKY45 plays a crucial role in benzothiadiazole-inducible blast resistance. Plant Cell.

[b31] Shimono M, Koga H, Akagi A (2012). Rice WRKY45 plays important roles in fungal and bacterial disease resistance. Mol. Plant Pathol.

[b32] Silverman P, Seskar M, Kanter D, Schweizer P, Metraux JP, Raskin I (1995). Salicylic acid in rice: biosynthesis, conjugation, and possible role. Plant Physiol.

[b33] Spoel SH, Mou Z, Tada Y, Spivey NW, Genschik P, Dong X (2009). Proteasome-mediated turnover of the transcription coactivator NPR1 plays dual roles in regulating plant immunity. Cell.

[b34] Sticher L, Mauch-Mani B, Métraux JP (1997). Systemic acquired resistance. Annu. Rev. Phytopathol.

[b35] Sugano S, Jiang C–J, Miyazawa S–I, Masumoto C, Yazawa K, Hayashi N, Shimono M, Nakayama A, Miyao M, Takatsuji H (2010). Role of OsNPR1 in rice defense program as revealed by genome-wide expression analysis. Plant Mol. Biol.

[b36] Tada Y, Spoel SH, Pajerowska-Mukhtar K, Mou Z, Song J, Wang C, Zuo J, Dong X (2008). Plant immunity requires conformational changes of NPR1 via *S*–nitrosylation and thioredoxins. Science.

[b301] Toki S, Hara N, Ono K, Onodera H, Tagiri A, Oka S, Tanaka H (2006). Early infection of scutellum tissue with Agrobacterium allows high speed transformation of rice. Plant J.

[b37] Valley CC, Metivier R, Solodin NM, Fowler AM, Mashek MT, Hill L, Alarid ET (2005). Differential regulation of estrogen-inducible proteolysis and transcription by the estrogen receptor α N terminus. Mol. Cell. Biol.

[b38] Vierstra RD (2009). The ubiquitin–26S proteasome system at the nexus of plant biology. Nat. Rev. Mol. Cell Biol.

[b39] Wang D, Amornsiripanitch N, Dong X (2006). A genomic approach to identify regulatory nodes in the transcriptional network of systemic acquired resistance in plants. PLoS Pathog.

[b40] Wu KL, Guo ZJ, Wang HH, Li J (2005). The WRKY family of transcription factors in rice and Arabidopsis and their origins. DNA Res.

[b41] Zou X, Seemann JR, Neuman D, Shen QJ (2004). A *WRKY* gene from creosote bush encodes an activator of the abscisic acid signaling pathway. J. Biol. Chem.

