# Supplementary material for: Nuclear ubiquitin proteasome degradation affects WRKY45 function in the rice defense program
Source: Plant J. 2012 Nov 8;73(2):302–13. doi: 10.1111/tpj.12035 (PMC3558880; doi:10.1111/tpj.12035)
Supplement: Supplementary file 1 [file tpj0073-0302-SD1.pptx]

## Slide 1
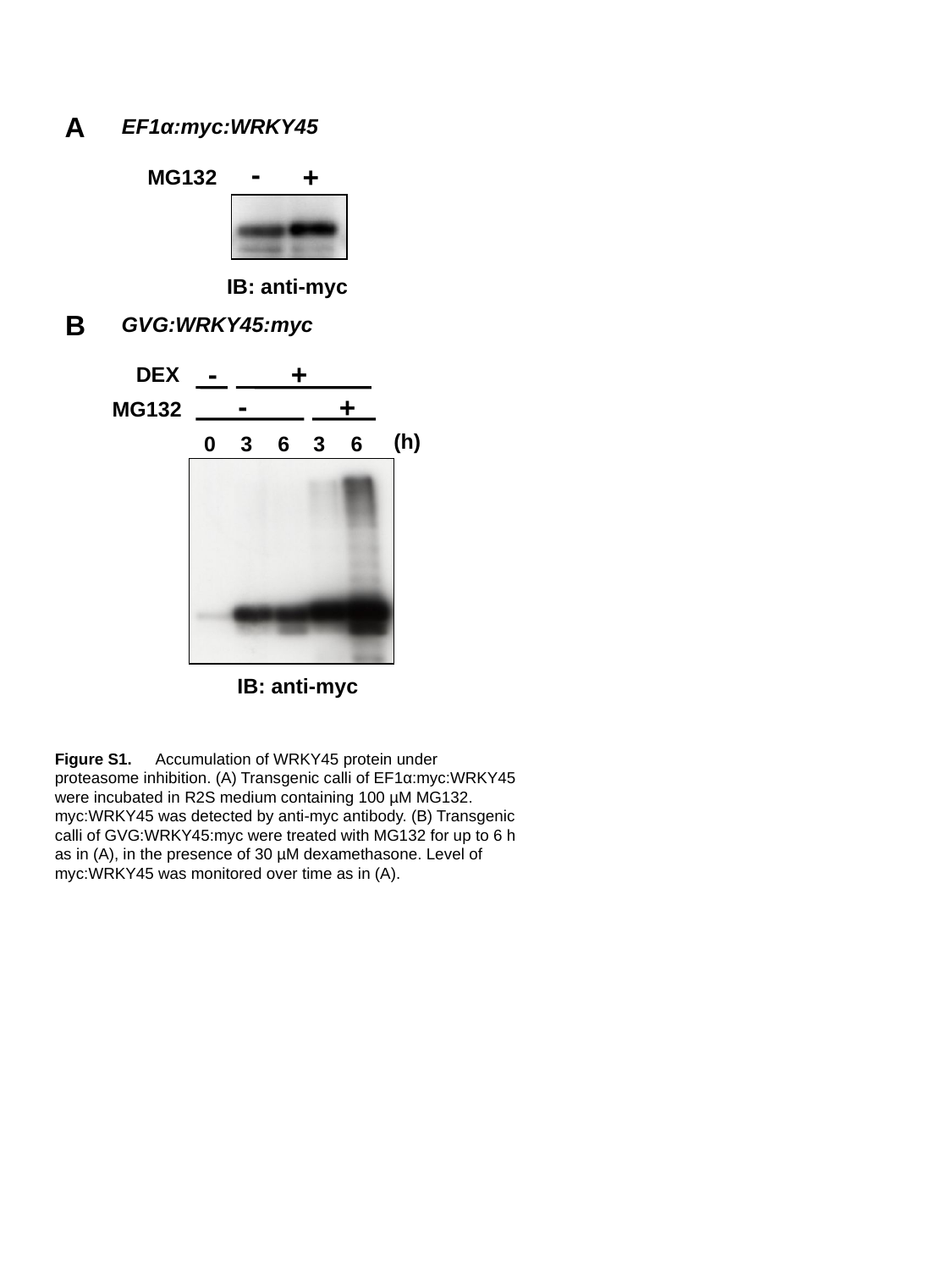

A
EF1α:myc:WRKY45
-
+
MG132
IB: anti-myc
B
GVG:WRKY45:myc
+
-
DEX
-
+
MG132
(h)
0
3
6
3
6
IB: anti-myc
Figure S1.　Accumulation of WRKY45 protein under proteasome inhibition. (A) Transgenic calli of EF1α:myc:WRKY45 were incubated in R2S medium containing 100 µM MG132. myc:WRKY45 was detected by anti-myc antibody. (B) Transgenic calli of GVG:WRKY45:myc were treated with MG132 for up to 6 h as in (A), in the presence of 30 µM dexamethasone. Level of myc:WRKY45 was monitored over time as in (A).
