# Supplementary material for: Nuclear ubiquitin proteasome degradation affects WRKY45 function in the rice defense program
Source: Plant J. 2012 Nov 8;73(2):302–13. doi: 10.1111/tpj.12035 (PMC3558880; doi:10.1111/tpj.12035)
Supplement: Supplementary file 2 [file tpj0073-0302-SD2.pptx]

## Slide 1
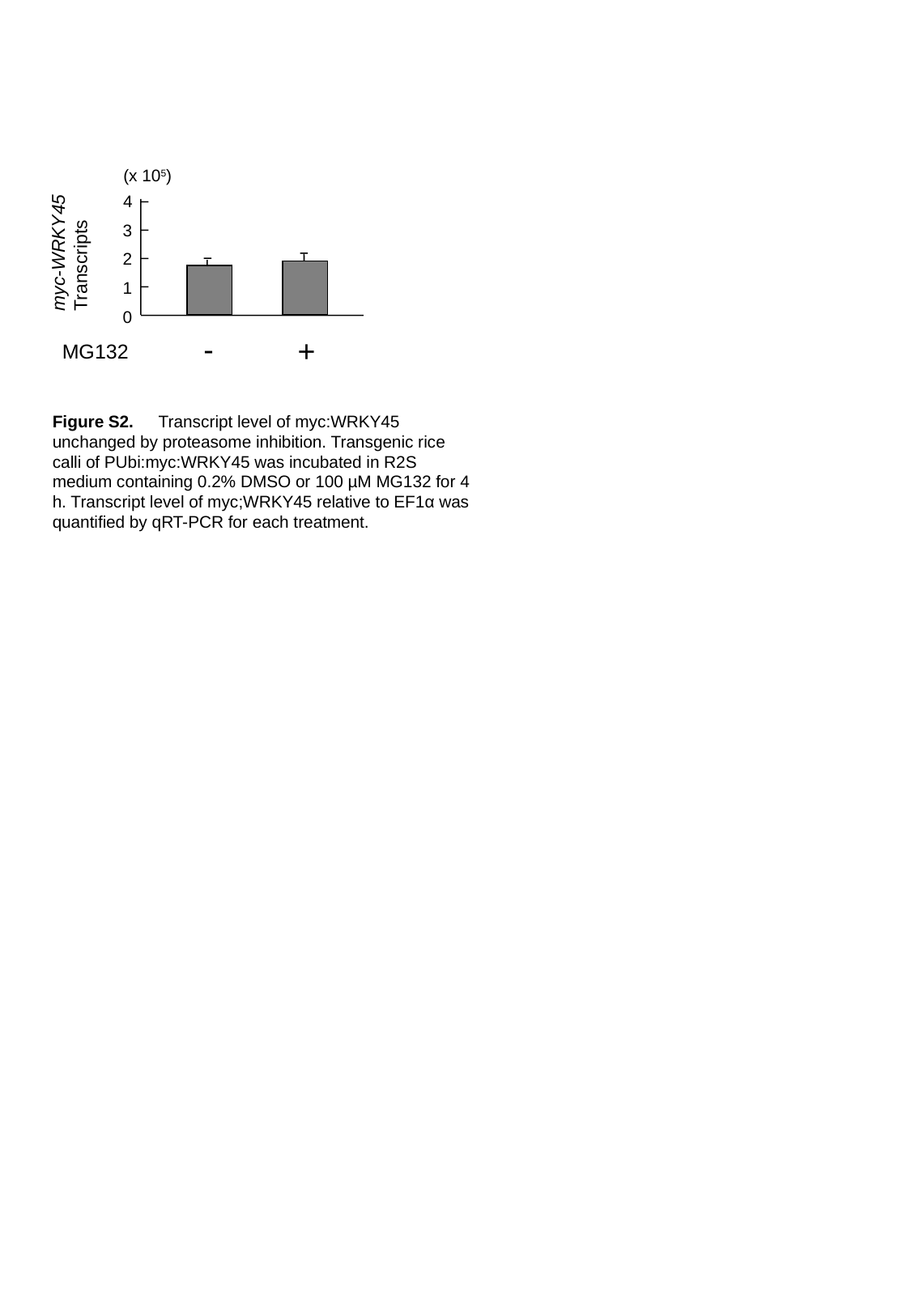

(x 105)
myc-WRKY45
Transcripts
4
3
2
1
0
-
+
MG132
Figure S2.　Transcript level of myc:WRKY45 unchanged by proteasome inhibition. Transgenic rice calli of PUbi:myc:WRKY45 was incubated in R2S medium containing 0.2% DMSO or 100 µM MG132 for 4 h. Transcript level of myc;WRKY45 relative to EF1α was quantified by qRT-PCR for each treatment.
