# Supplementary material for: Nuclear ubiquitin proteasome degradation affects WRKY45 function in the rice defense program
Source: Plant J. 2012 Nov 8;73(2):302–13. doi: 10.1111/tpj.12035 (PMC3558880; doi:10.1111/tpj.12035)
Supplement: Supplementary file 3 [file tpj0073-0302-SD3.pptx]

## Slide 1
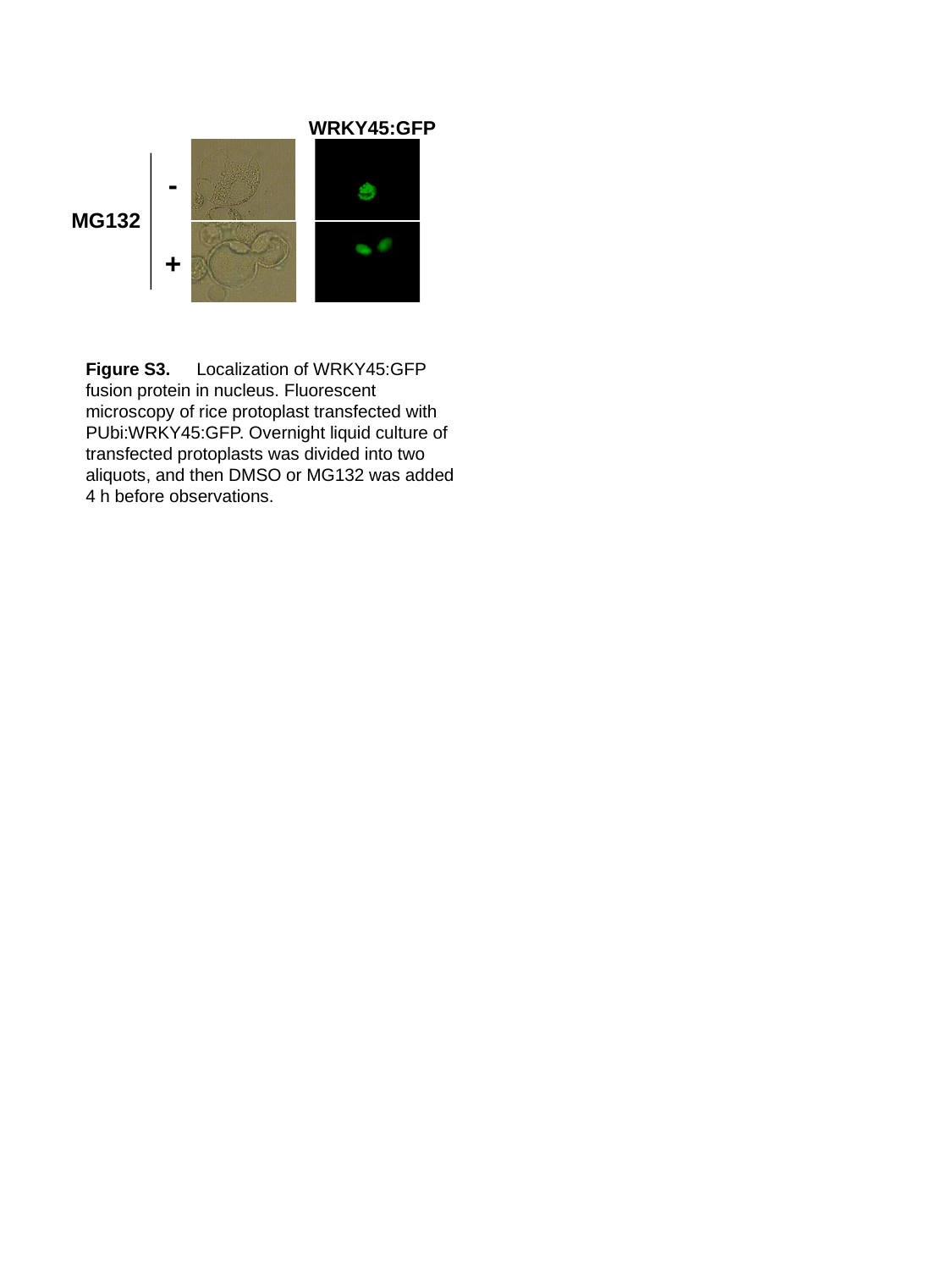

WRKY45:GFP
-
MG132
+
Figure S3.　Localization of WRKY45:GFP fusion protein in nucleus. Fluorescent microscopy of rice protoplast transfected with PUbi:WRKY45:GFP. Overnight liquid culture of transfected protoplasts was divided into two aliquots, and then DMSO or MG132 was added 4 h before observations.
