# Supplementary material for: Nuclear ubiquitin proteasome degradation affects WRKY45 function in the rice defense program
Source: Plant J. 2012 Nov 8;73(2):302–13. doi: 10.1111/tpj.12035 (PMC3558880; doi:10.1111/tpj.12035)
Supplement: Supplementary file 4 [file tpj0073-0302-SD4.pptx]

## Slide 1
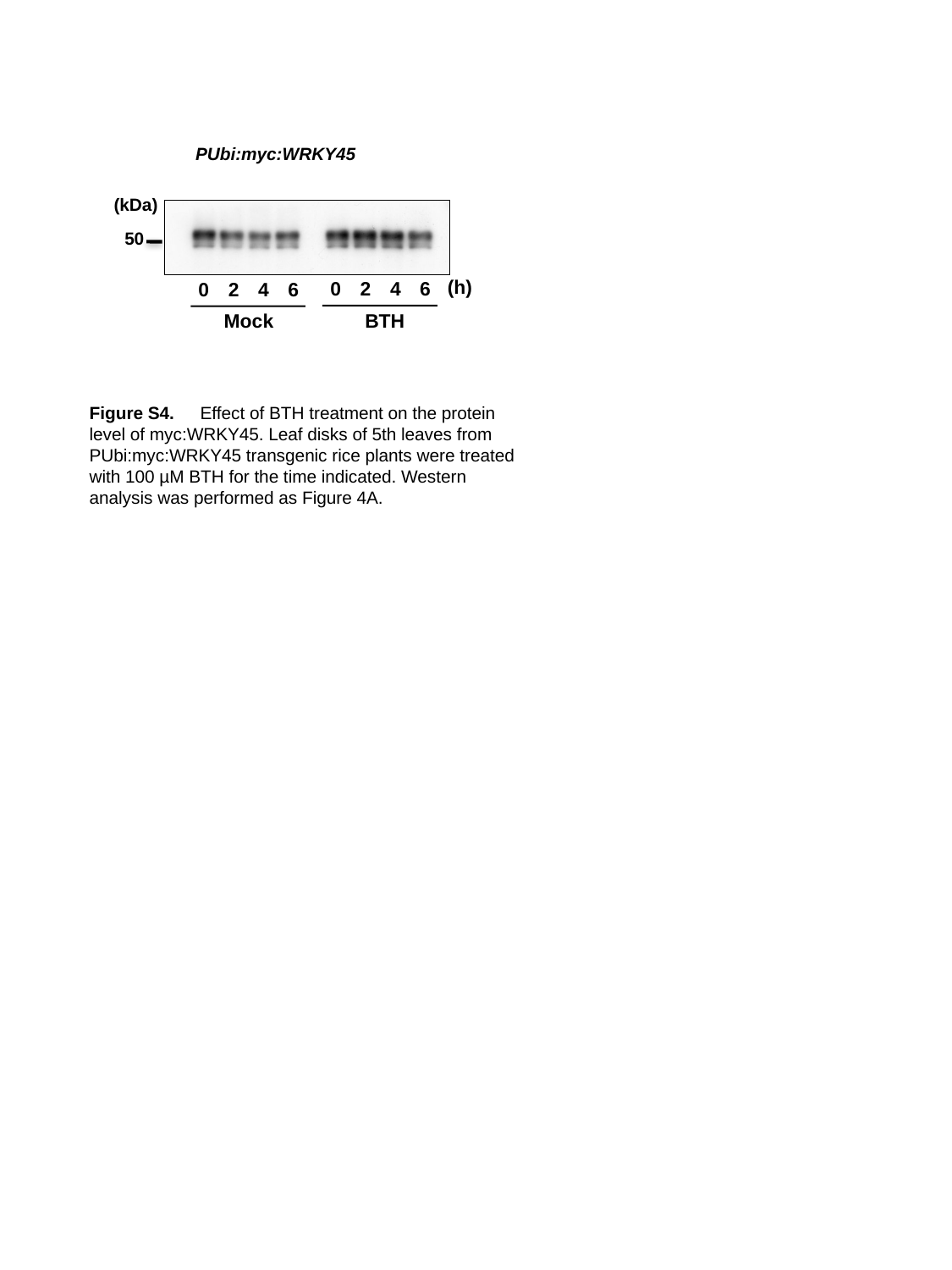

PUbi:myc:WRKY45
(kDa)
50
(h)
0
2
4
6
0
2
4
6
Mock
BTH
Figure S4.　Effect of BTH treatment on the protein level of myc:WRKY45. Leaf disks of 5th leaves from PUbi:myc:WRKY45 transgenic rice plants were treated with 100 µM BTH for the time indicated. Western analysis was performed as Figure 4A.
