# Supplementary material for: Nuclear ubiquitin proteasome degradation affects WRKY45 function in the rice defense program
Source: Plant J. 2012 Nov 8;73(2):302–13. doi: 10.1111/tpj.12035 (PMC3558880; doi:10.1111/tpj.12035)
Supplement: Supplementary file 5 [file tpj0073-0302-SD5.pptx]

## Slide 1
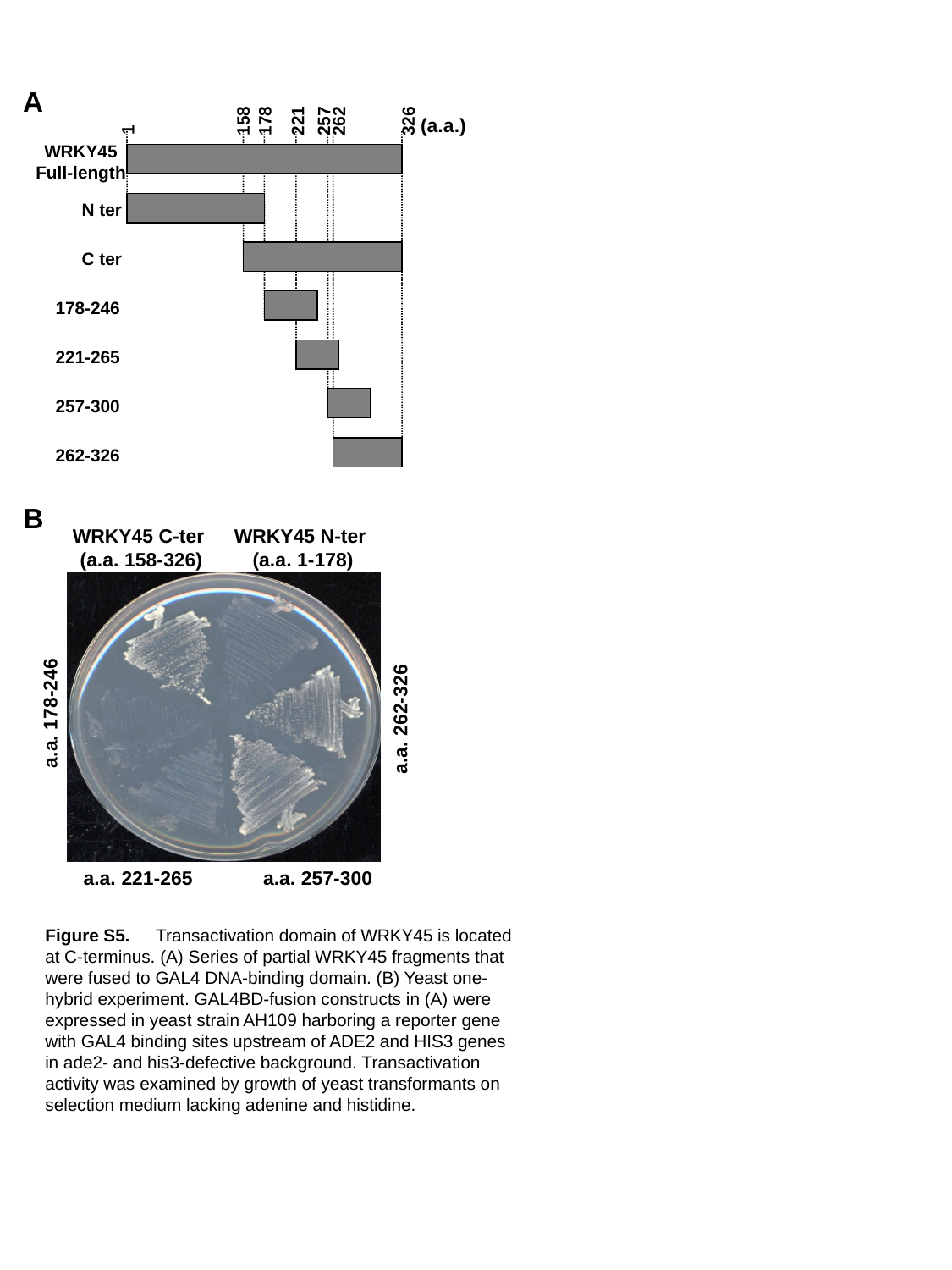

A
158
178
221
257
262
326
(a.a.)
1
WRKY45
Full-length
N ter
C ter
178-246
221-265
257-300
262-326
B
WRKY45 C-ter
(a.a. 158-326)
WRKY45 N-ter
(a.a. 1-178)
a.a. 178-246
a.a. 262-326
a.a. 221-265
a.a. 257-300
Figure S5.　Transactivation domain of WRKY45 is located at C-terminus. (A) Series of partial WRKY45 fragments that were fused to GAL4 DNA-binding domain. (B) Yeast one-hybrid experiment. GAL4BD-fusion constructs in (A) were expressed in yeast strain AH109 harboring a reporter gene with GAL4 binding sites upstream of ADE2 and HIS3 genes in ade2- and his3-defective background. Transactivation activity was examined by growth of yeast transformants on selection medium lacking adenine and histidine.
