# Supplementary material for: Nuclear ubiquitin proteasome degradation affects WRKY45 function in the rice defense program
Source: Plant J. 2012 Nov 8;73(2):302–13. doi: 10.1111/tpj.12035 (PMC3558880; doi:10.1111/tpj.12035)
Supplement: Supplementary file 6 [file tpj0073-0302-SD6.pptx]

## Slide 1
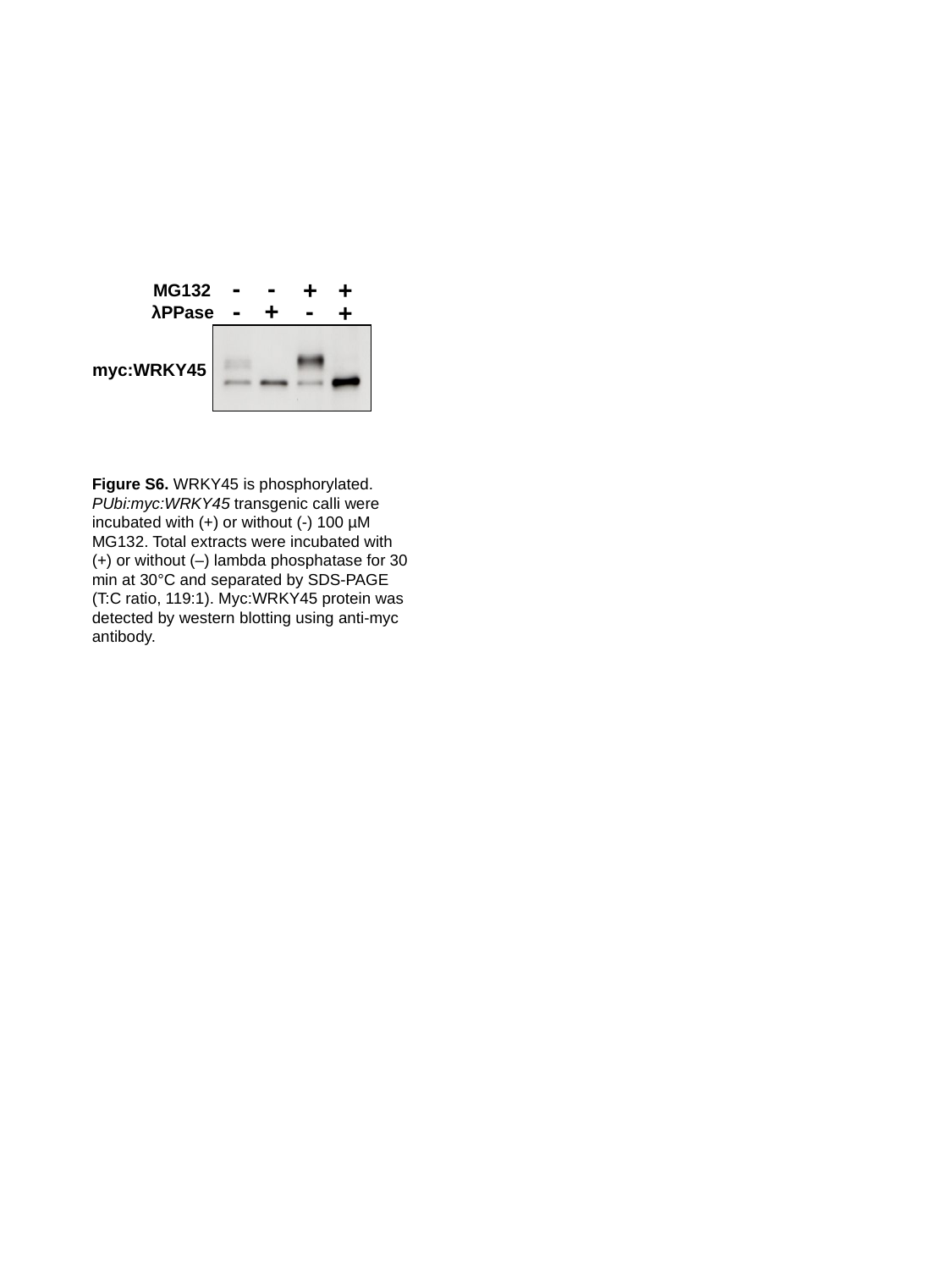

-
-
+
+
MG132
-
-
+
+
λPPase
myc:WRKY45
Figure S6. WRKY45 is phosphorylated.
PUbi:myc:WRKY45 transgenic calli were incubated with (+) or without (-) 100 µM MG132. Total extracts were incubated with (+) or without (–) lambda phosphatase for 30 min at 30°C and separated by SDS-PAGE (T:C ratio, 119:1). Myc:WRKY45 protein was detected by western blotting using anti-myc antibody.
