# Supplementary material for: Nuclear ubiquitin proteasome degradation affects WRKY45 function in the rice defense program
Source: Plant J. 2012 Nov 8;73(2):302–13. doi: 10.1111/tpj.12035 (PMC3558880; doi:10.1111/tpj.12035)
Supplement: Supplementary file 7 [file tpj0073-0302-SD7.docx]

**Table S1.** Primers used for qPCR.

| Gene | Primer name | Primer Sequence (5’🡪3’) |
| --- | --- | --- |
| *Actin1* | ACT1-RTF | TCCATCTTGGCATCTCTCAG |
|  | ACT1-RTR | GTACCCGCATCAGGCATCTG |
| *WRKY45* | W45-RTF | CGGGTAAAACGATCGAAAGA |
|  | W45-RTR | TTTCGAAAGCGGAAGAACAG |
| *WRKY62* | W62-RTF | CACACTCGACCTGACGAACC |
|  | W62-RTR | ACTTGCACCACCTCCTCCAC |
| *GSTU4* | GST-RTF | TCCCATTGTTTGGGAGAAAA |
|  | GST-RTR | GAATATTCCTTGCATTATTCAGACA |
| *LOX* | LOX-RTF | CCGTACCAGCTGATGAAGC |
|  | LOXRTR | TTTTGGAGCGTTTTGTCTCA |
